# Survey datasets on patterns of utilization of mental healthcare services among people living with mental illness

**DOI:** 10.1016/j.dib.2018.06.086

**Published:** 2018-07-05

**Authors:** Tomike I. Olawande, Hilary I. Okagbue, Ayodele S. Jegede, Patrick A. Edewor, Lukman T. Fasasi

**Affiliations:** a Department of Sociology, Covenant University, Ota, Nigeria; b Department of Mathematics, Covenant University, Ota, Nigeria; c Department of Sociology, University of Ibadan, Ibadan, Nigeria

**Keywords:** Survey, Utilization questionnaire, Survey analytics, Statistics, Mental health, Psychiatry

## Abstract

The data was obtained from a field survey aimed at measuring the patterns of utilization of mental healthcare services among people living with mental illness. The data was collected using a standardized and structured questionnaire from People Living with Mental Illness (PLMI) receiving treatment and the care-givers of People Living with Mental Illness. Three psychiatric hospitals in Ogun state, Nigeria were the population from which the samples were taken. Chi-square test of independence and correspondence analysis were used to present the data in analyzed form.

**Specification Table**

**Table:** 

Subject Area	Psychology
More Specific subject area	Quantitative Psychology and Mental Health
Type of data	Table and text file
How data was acquired	Field survey
Data format	Raw, partial analyzed
Experimental factors	Pattern of utilization of mental healthcare services
Experimental features	Only those receiving treatments and the care-givers (in the case of very unstable patients) were considered. Also only those residents in the study areas were considered. Adults younger than 18 years were also excluded.
Data Source location	Covenant University Sociology Laboratory, Ota, Nigeria
Data accessibility	All the data are in this data article

**Significance of the data**

• The central theme is the study of utilization of mental healthcare facilities among people living with mental illness.
• The data could be useful in monitoring the extent to which the mental health services are available and utilized.
• The study can be replicated to other countries with similar demographic factors.
• The data can be used in the overall study of mental health.

## 1 Data

The data is a summary of responses from a field survey. Structured questionnaires were administered to People Living with Mental Illness (PLWMI) and their caregivers and the aim is to measure the patterns of utilization of mental healthcare services among PLWMI.

Only those receiving treatments and the care-givers (in the case of very unstable patients) were considered. Also, those residents in the study areas that are of Yoruba origin were considered. Adults younger than 18 years were excluded from the study.

**Fig. 1:**
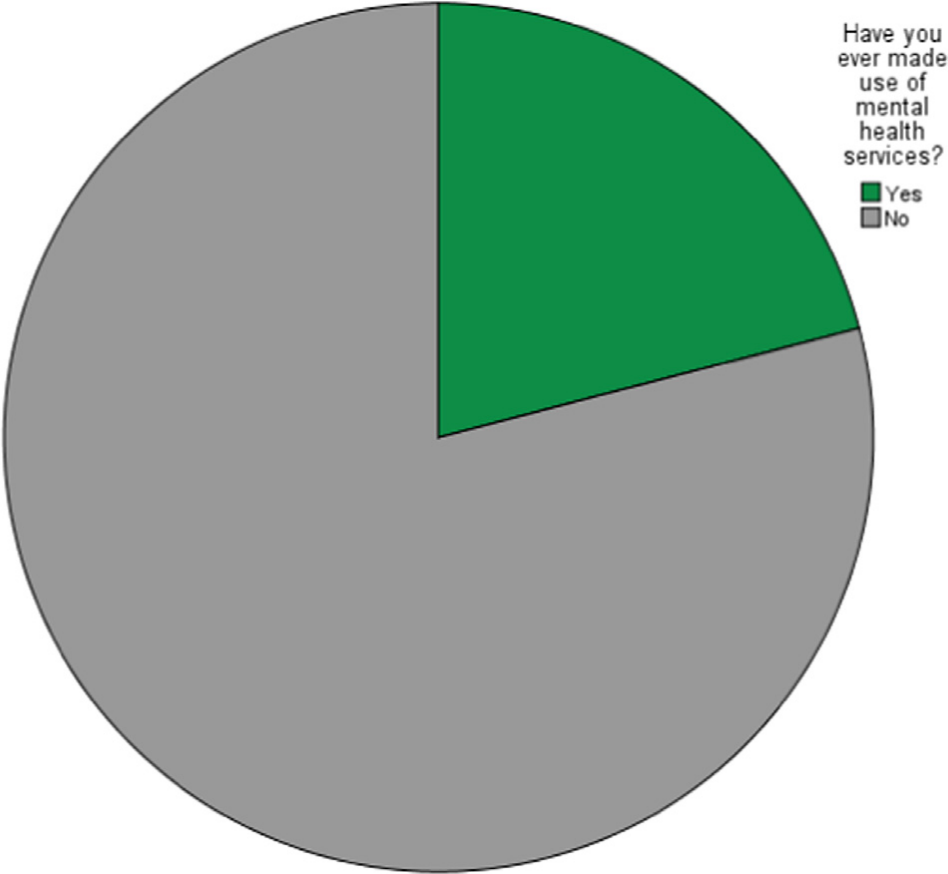
Perceived use of the mental healthcare services by the respondents.

**Fig. 2:**
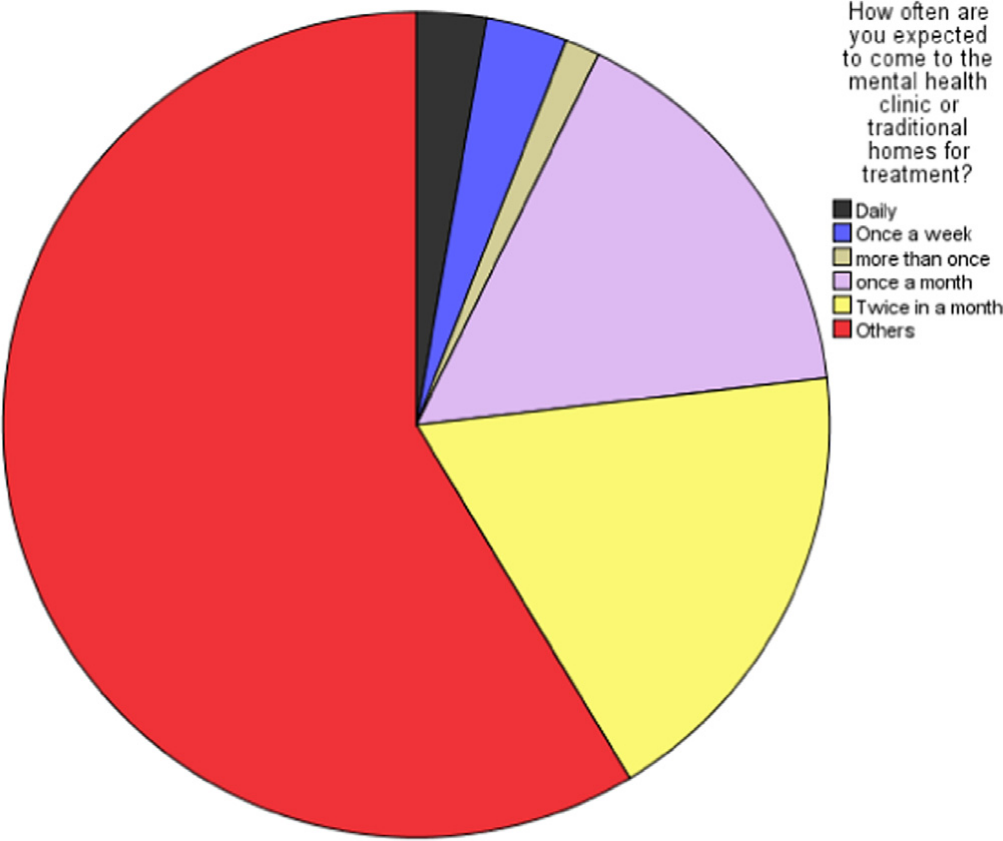
Perceived frequency of use of the mental healthcare services by the respondents.

**Fig. 3:**
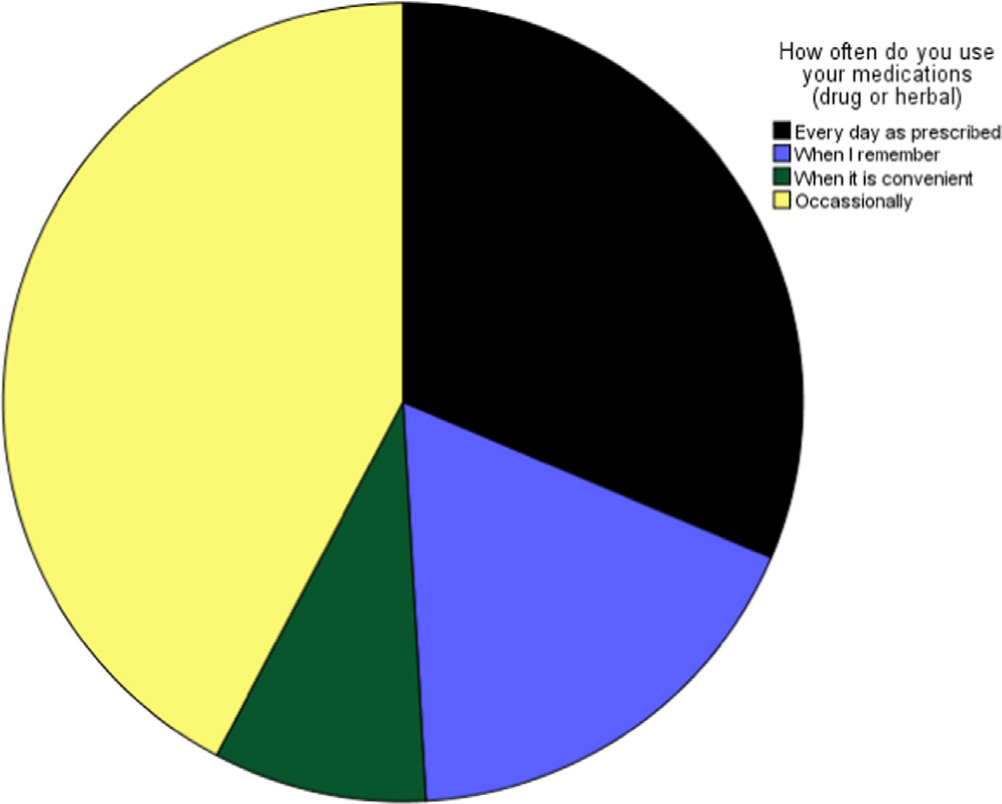
Frequency of taking prescribed medications.

**Fig. 4:**
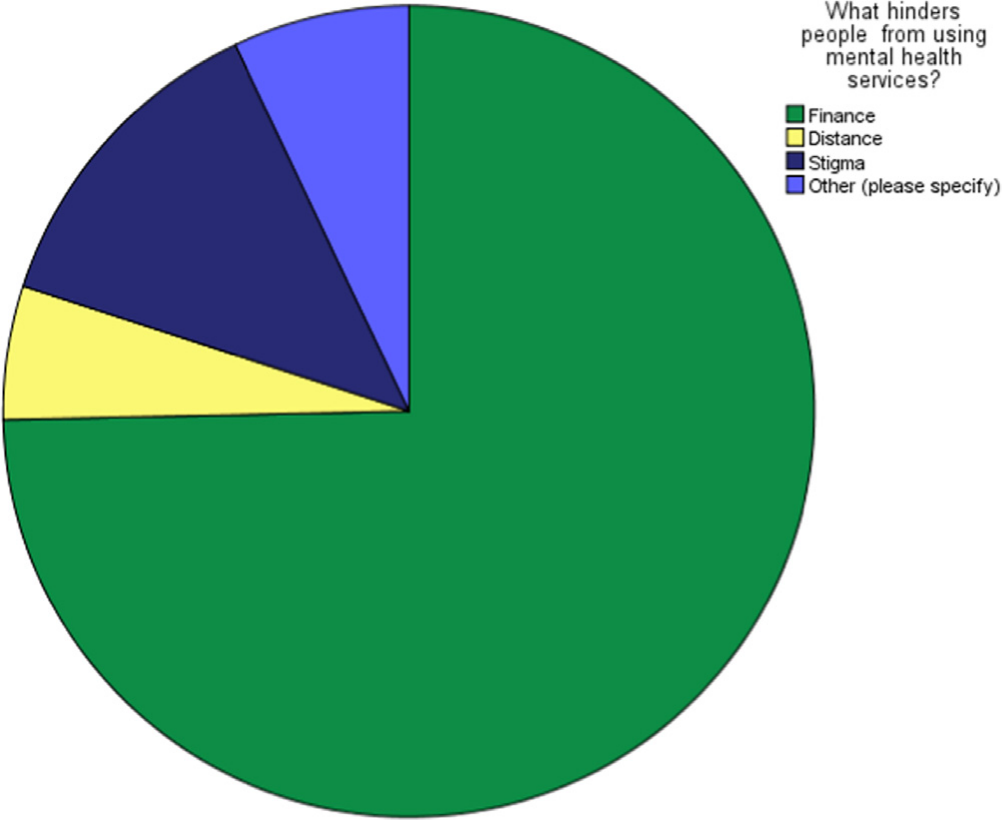
Perceived obstacle of using the available mental healthcare services.

The pattern of utilization mental healthcare services in this context was determined by the perceived use of the mental healthcare services by the respondents, frequency of use, frequency of taking prescribed medications and the perceived obstacle of using the available mental healthcare services. These are shown in Fig. 1, Fig. 2, Fig. 3, Fig. 4. The raw data can be assessed as Supplementary data 1 and the questionnaire can be assessed as Supplementary data 2.

## 2 Experimental design, materials and methods

Mental illness has been believed by numerous experts to be caused amongst others by depression, alcohol and substance abuse, stress, violence against women or minors, post-traumatic stress disorder, women׳s infertility and biological factors. Mental health in particular requires special help, care and management. The treatment may come as psychotherapy and medications which are available in mental healthcare services. The availability of mental health services determines their patterns of usage or utilization [1], [2], [3], [4], [5].

Utilization is connected with ease of use, excellence service, good customer relations, affordable fees charge, management and socio-economic factors.

Questionnaire was used in this article to measure the pattern of utilization of mental healthcare services in Psychiatric hospitals located in three local Government areas of Ogun state, Nigeria. The utilization of the mental healthcare services in the demographics of the study area in particular and Nigeria in general are historically low due to long distance, unavailability of medications, stigmatization, epileptic or skeletal services, poor road networks, poverty and dearth of skilled psychiatrics [6], [7], [8], [9], [10], [11], [12], [13], [14], [15]. Generally, the following statistical analysis and survey methods in these articles can be useful [16], [17], [18], [19], [20], [21], [22], [23], [24], [25], [26], [27], [28], [29], [30].

### 2.1 Contingency analysis

Chi-square test of independence was used to determine the association between the measure of utilization of mental healthcare services and the socio-demographics of the respondents and is presented in Table 1, Table 2.

**Table 1:** Contingency analysis between the usage of mental services and the soocio-demographic variables.

Socio-demographic factors	Chi-square	P value
Gender	0.153316	0.695387
Age	5.595044	0.347636
Marital status	12.941725	0.023931
Religion	2.046284	0.562856
Level of education	7.503471	0.483409
Occupation/ Profession	12.302178	0.138222
Income	3.307660	0.507719
Duration of residency in the studied area	5.069540	0.407453
Family type	2.222229	0.329192
Form of marriage	1.108207	0.574587

**Table 2:** Contingency analysis between the perceived hindrance of mental services and the soocio-demographic variables.

Socio-demographic factors	Chi-square	P value
Gender	2.667740	0.445737
Age	40.262166	0.000414
Marital status	20.179331	0.165161
Religion	6.378052	0.701566
Level of education	32.969706	0.104714
Occupation/ Profession	31.410814	0.142287
Income	7.675522	0.809946
Duration of residency in the studied area	20.965525	0.137934
Family type	7.046988	0.316524
Form of marriage	4.321635	0.633238

**Remarks**: P-value less than 0.05 imply association.

### 2.2 Correlational analysis

The correlational studies are important to reveal the strength and nature of the observed linear relationship that exist between the measure of utilization and the socio-demographic variables. These are presented in Table 3, Table 4.

**Table 3:** Correlational analysis between the usage of mental services and the soocio-demographic variables.

Socio-demographic factors	Pearson׳s R	P value
Gender	-0.012084	0.695721
Age	0.026051	0.399072
Marital status	-0.088972	0.003910
Religion	-0.017942	0.561412
Level of education	-0.037189	0.228575
Occupation/ Profession	-0.030210	0.328092
Income	-0.036221	0.240918
Duration of residency in the studied area	0.015793	0.609235
Family type	-0.009194	0.766027
Form of marriage	-0.006283	0.838854

**Table 4:** Correlational analysis between the perceived hindrance of mental services and the soocio-demographic variables.

Socio-demographic factors	Pearson׳s R	P value
Gender	-0.001236	0.968093
Age	0.078624	0.010815
Marital status	-0.032353	0.294916
Religion	0.002405	0.937959
Level of education	-0.025976	0.400421
Occupation/ Profession	-0.038578	0.211642
Income	0.018045	0.559159
Duration of residency in the studied area	0.028956	0.348574
Family type	0.037799	0.221030
Form of marriage	-0.032385	0.294450

### 2.3 Correspondence analysis

Correspondence analysis is performed to visually display the contributions of the income of the respondents to the hindrance from using mental health services. Details on correspondence analysis can be found in [31], [32], [33], [34], [35].

**Fig. 5:**
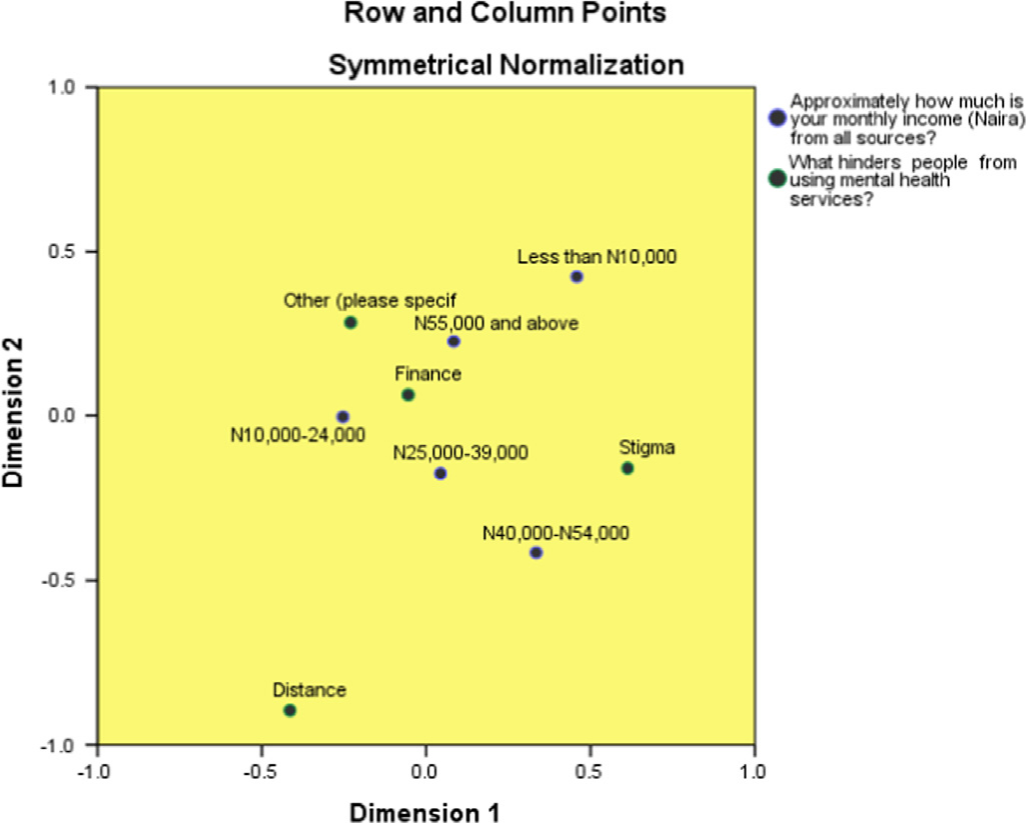
Biplot showing the perceived relationship in graphical form.

The results are presented as follows: Correspondence table (Table 5), model summary (Table 6), overview row points (Table 7), overview column points (Table 8) and biplot (Fig. 5).

**Table 5:** Correspondence table of patterns of utilization of mental healthcare services among people living with mental illness.

What hinders people from using mental health services?	Approximately how much is your monthly income (Naira) from all sources?					
	Less than N10,000	N10,000–24,000	N25,000–39,000	N40,000-N54,000	N55,000 and above	Active Margin
Finance	68	346	100	107	163	784
Distance	2	27	8	10	9	56
Stigma	14	50	18	25	29	136
Other (please specify)	6	34	8	9	17	74
Active Margin	90	457	134	151	218	1050

**Table 6:** model summary of patterns of utilization of mental healthcare services among people living with mental illness.

Dimension	Singular Value	Inertia	Chi Square	Sig.	Proportion of Inertia		Confidence Singular Value	
					Accounted for	Cumulative	Standard Deviation	Correlation
								2
1	0.064	0.004			0.557	0.557	0.031	-0.098
2	0.055	0.003			0.411	0.968	0.029	
3	0.015	0.000			0.032	1.000		
Total		0.007	7.676	0.810^a^	1.000	1.000		

The p value indicates that the income of the respondents is not associated with the hindrance they encountered in the utilization of mental healthcare services.

a 12 degrees of freedom

**Table 7:** Overview row points table of patterns of utilization of mental healthcare services among people living with mental illness.

What hinders people from using mental health services?	Mass	Score in Dimension		Inertia	Contribution				
		1	2		Of Point to Inertia of Dimension		Of Dimension to Inertia of Point		
					1	2	1	2	Total
Finance	.747	-.055	.065	.000	.035	.057	.409	.484	.893
Distance	.053	-.415	-.896	.003	.144	.780	.199	.799	.998
Stigma	.130	.613	-.158	.003	.762	.059	.943	.054	.997
Other (please specify)	.070	-.230	.285	.001	.059	.104	.326	.428	.755
Active Total	1.000			.007	1.000	1.000

**Table 8:** Overview column points table of patterns of utilization of mental healthcare services among people living with mental illness.

Approximately how much is your monthly income (Naira) from all sources?	Mass	Score in Dimension		Inertia	Contribution				
		1	2		Of Point to Inertia of Dimension		Of Dimension to Inertia of Point		
					1	2	1	2	Total
Less than N10,000	.086	.458	.424	.002	.282	.281	.566	.416	.981
N10,000–24,000	.435	-.254	-.003	.002	.439	.000	1.000	.000	1.000
N25,000–39,000	.128	.044	-.174	.000	.004	.071	.048	.644	.691
N40,000-N54,000	.144	.334	-.416	.002	.252	.453	.426	.565	.991
N55,000 and above	.208	.084	.227	.001	.023	.195	.124	.780	.905
Active Total	1.000			.007	1.000	1.000			

a. Symmetrical normalization

**Remarks:** The data was explained by two dimensions. Distance seems not to be perceived hindrance to utilization of mental healthcare services in the studied area.
