# Supplementary material for: Survey datasets on patterns of utilization of mental healthcare services among people living with mental illness
Source: Data Brief. 2018 Jul 5;19:2095–103. doi: 10.1016/j.dib.2018.06.086 (PMC6141371; doi:10.1016/j.dib.2018.06.086)
Supplement: Supplementary file 3 — Supplementary material [file mmc3.docx]

**
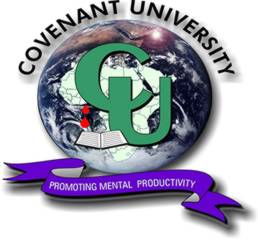
**

**Short Questionnaire**

Department of Sociology

College of Business and Social Sciences

Covenant University, Ota, Ogun State

Dear Sir/Madam,

Questionnaire No……………

This questionnaire is designed to obtain information on use of mental healthcare services. I assure you that the information provided will be used solely for research and academic purposes without any record of your identity.

Thank you for your anticipated cooperation and prompt response.

**SECTION A: BASIC DATA**

1. Gender: (a) Male ( ) (b) Female ( ).
2. Age: (a) 20-24 years ( ) (b) 25-29 years ( ) (c) 30-34 years ( ) (d) 35-39 years ( ) (e) 40-44 years ( ) (f) 45 years and above ( )
3. Marital Status: (a) Single ( ) (b) Married ( ) (c) Separated ( ) (d) Divorce ( ) (e) Widowed ( ) (f) Others ( )
4. Religion: (a) Christianity ( ) (b) Islam ( ) (c) Traditional ( ) (d) Others ( )
5. Highest Current Level of Educational status: (a) No education ( ) (b) Quaranic education ( ) (c) Primary education ( ) (d) Junior secondary education ( ) (e) Senior secondary education ( ) (f) Ordinary National Diploma ( ) (g) Higher National Diploma/ University Degree () (h) Postgraduate Degree ( ) (i) National Certificate of Education
6. Occupation/ Profession (a) Unemployed ( ) (b) Student ( ) (c) Apprentice ( ) (d) Civil servant ( ) (e) Teaching ( ) (f) Business ( ) (g) Petty trading ( ) (h) Farming ( ) (g) Artisanship ( ).
7. Monthly income in Naira (a) Less than 10,000 ( ) (b) 11,000-24,000 ( ) (c) 25,000-39000 ( ) (d) 40,000-54,000 ( ) (e) Above 55000 ( ).
8. How long have you been living in this community? (a) Less than 10 years ( ) (b) 10-14 years ( ) (c) 15-19 years ( ) (d) 20-24 years ( ) (e) 25-29 years ( ) (f) 30 years and Above ( ).
9. What is your current family type? (a) Nuclear ( ) (b) Extended ( ) (c) Others ( ).
10. What is your current form of marriage? (a) Monogamy ( ) (b) Polygyny ( ) (c) Others ( ).

**SECTION B**

1. Have you ever made use of mental health services? (a) Yes ( ) (b) No ( ) **If no, go to 14.**
2. How often are you expected to come to the mental health clinic or traditional homes for treatment? (a) Daily ( ) (b) Once in a week ( ) (c) More than once in a week ( ) (d) Once a month ( ) (e) Twice a month ( ) (f) Others ( )
3. How often do you use your medications (drugs or herbal)? (a) Everyday as prescribed ( ) (b)when I remember ( ) (c) When it is convenient ( ) (d) Occasionally ( )
4. What hinders people from using mental health services? (a) Finance ( ) (b)Distance ( ) (c) Stigma ( ) (d) Other ( )
